# Combining agent based-models and virtual screening techniques to predict the best citrus-derived vaccine adjuvants against human papilloma virus

**DOI:** 10.1186/s12859-017-1961-9

**Published:** 2017-12-28

**Authors:** Marzio Pennisi, Giulia Russo, Silvia Ravalli, Francesco Pappalardo

**Affiliations:** 1 Department of Mathematics and Computer Science, University of Catania, 95125 Catania, Italy; 2 Department of Biomedical and Biotechnological Sciences, University of Catania, 95123 Catania, Italy; 3 Department of Drug Sciences, University of Catania, 95125 Catania, Italy

**Keywords:** Multi agent systems, vaccines, Adjuvants, Virtual screening, HPV

## Abstract

**Background:**

Human papillomavirus infection is a global social burden that, every year, leads to thousands new diagnosis of cancer. The introduction of a protocol of immunization, with Gardasil and Cervarix vaccines, has radically changed the way this infection easily spreads among people. Even though vaccination is only preventive and not therapeutic, it is a strong tool capable to avoid the consequences that this pathogen could cause. Gardasil vaccine is not free from side effects and the duration of immunity is not always well determined. This work aim to enhance the effects of the vaccination by using a new class of adjuvants and a different administration protocol. Due to their minimum side effects, their easy extraction, their low production costs and their proven immune stimulating activity, citrus-derived molecules are valid candidates to be administered as adjuvants in a vaccine formulation against Hpv.

**Results:**

With the aim to get a stronger immune response against Hpv infection we built an in silico model that delivers a way to predict the best adjuvants and the optimal means of administration to obtain such a goal. Simulations envisaged that the use of Neohesperidin elicited a strong immune response that was then validated in vivo.

**Conclusions:**

We built up a computational infrastructure made by a virtual screening approach able to preselect promising citrus derived compounds, and by an agent based model that reproduces HPV dynamics subject to vaccine stimulation. This integrated methodology was able to predict the best protocol that confers a very good immune response against HPV infection. We finally tested the in silico results through in vivo experiments on mice, finding good agreement.

## Background

Human papillomavirus (Hpv) is a member of the Papovaviridae family, a successful infectious group of small, non-lytic, non-enveloped viruses with over 180 genotypes identified. Hpv infection has become the most common sexually transmitted disease all over the world, because of its peculiar mechanism to easily escape the immune system; it also represents a global social burden that, every year, leads to thousands new diagnosis of cancer [1, 2]. Globally, around 500,000 women are diagnosed with cervical cancer every year and more than half die because of that. High risk countries include Eastern and Southern Africa, Melanesia, South America, South-Central Asia and Eastern Europe [3, 4].

The concern about the risk of this type of infection regards two main factors: firstly, it deals with an infective agent that could lead to cancer development; secondly, because of social reasons, the highest risk individuals are represented by very young women who could experience a traumatic disease. Besides common risk factors of infection, like early first sexual intercourse and multiple sexual partners, there are a lot of factors linked to persistence. Some of them are history of genital neoplasia (vaginal, vulvar, anal), tobacco use, immune suppression, co-infection with other pathogens and long-term use of oral contraceptives [5].

Papillomaviruses are species and tissue specific, they penetrate and infect the pluristratified squamous epithelium of the cervix, if microwounds are present (e.g. microtrauma that exposes the basement membrane). The infection takes place at the basal layer, which is the lowest part of the epithelium. The keratinocytes (Kcs), which represent most of the cells of the basal layer, are the main target of Hpv. Although they predominantly belong to the epidermis rather than to the immune system machinery, they play an important role as innate immune system tools: they act as non-professional Antigen Presenting Cells (APC), being able to present peptides in association with MHC I/II [6, 7]; they are able to secrete pro-inflammatory cytokines and chemokines (IL-1, IL-6, IL-10, IL-18, TNF) and can express Toll-like receptors (TLR), located both on cell surface (TLR1, TLR2, TLR4, TLR5, TLR6) and in endosomes (TLR3 and TLR9).

Hpv has developed and improved several mechanisms to avoid both initial recognition and adaptive immunity [8, 9]. The key is to maintain a low profile: infection occurs at the basal layer of epidermis but the virus increases his replicative cycle, exclusively, when Kcs exit the basement membrane to differentiate; since the upper layers have poor access to vascular and lymphatic channels, it is given to the virus a good chance to stay away from the immune effectors. Since there is not a specific therapy yet to treat human papillomavirus correlated carcinoma, vaccination remains the best way to avoid this disease by preventing the infection [10].

In 2006, the EMA authorized the first vaccine for Hpv types 16 and 18, responsible for 70% of cervix carcinoma cases, and 6 and 11 Hpv types, main cause of genital warts. The first studies about this vaccine followed the discovery, in 1993, that L1 proteins (the major capsid protein responsible for virion assembly and DNA packaging) may be assembled as VLPs, virus-like particles. These entities resemble natural virus but are not infectious. Since they do not contain viral genetic material but maintain their immunogenic properties, they can be administered in a vaccination protocol [11]. The interest grew when the selfassembly event, that leads to these particles, was found even in vitro. On this basis, two anti-Hpv vaccines have been developed: Gardasil and Cervarix. The introduction of a protocol of immunization, from 2006, with Gardasil and Cervarix vaccines, has radically changed the way this infection easily spreads among people [12, 13]. Even though vaccination is only preventive and not therapeutic, it is a strong tool to avoid the consequences that this pathogen could cause. Indeed, vaccines still are the best way to prevent infectious disorders. Nowadays, several novel and risk-free vaccines have been designed: the use of well-identified antigens represents a mandatory requirement in terms of safety within the vaccine development process. Subunit preparations represent a valid alternative to live formulations especially for pregnant women, people who are immunocompromised or suffer of chronic illnesses.

Unfortunately, subunit antigens are inadequate immunogens when administered alone, they require two or more doses and need to be administered in specific periods of time to effectively immunize against the pathogen. Some viruses contain different structural proteins and their identification is not always simple; other important steps to take into consideration are protein purification and production in large scale that own typical issues [14].

For what concerns specific vaccines targeting Hpv, such as Gardasil and Cervarix, they are not free from side effects and in particular, for the case of Gardasil, the duration of immunity is not well determined [15].

Furthermore, Cervarix does not provide protection to all Hpv types or to women previously exposed to the virus through sexual activity; this is also the reason why it should be recommended at early age [16]. To potentiate the immune response, additional molecules called adjuvants are generally included within the vaccine formulation. Several molecules are employed as adjuvants and could be useful to boost or protract the immunogenicity; they are also able to reduce the number of injections, or the antigen dose and to improve immunization in high risk population. Adjuvants may switch immune system response to T helper 1 (Th1) or T helper 2 (Th2) type and could also overcome technological problems through two possibilities: one involves the possibility to uptake antigens inside the adjuvants that then can be used as delivery system; the other one behaves as a depot substance to protect the antigens and modulate a controlled release of the entire vaccine formulation.

Adjuvants are divided into two main categories: the first one deals with molecules enhancing the processing of vaccine antigens by APC. For example, mineral salts, emulsions, liposomes and virosomes. The second group includes immunostimulant entities, like cytokines, Toll-like receptor agonists and saponines that meliorate the immune responses towards specific agents by promoting the releasing of cytokines [17].

Aluminum adjuvants are largely used to enhance vaccine immunogenicity through stimulation of high antibody titers. Among the three main forms of aluminum adjuvants, aluminum phosphate (AlPO(4)), aluminum hydroxide (AlOH) and amorphous aluminum hydroxyphosphate sulfate (AAHS), the latter takes part of Gardasil formulation, as best choice. In over 70 years, aluminum adjuvants have demonstrated safety and efficacy in combination with vaccine formulation and remains the most accepted adjuvants for human vaccines [18, 19].

Even though a lot of new adjuvants have already been investigated and tested (e.g., lipopeptides, polysaccharides, nucleic acids, emulsions, cytokines, detoxified toxins and mineral salts), very few of them have been approved and, currently, take part of modern formulation [20–22].

Moreover, phytosterols represent another important class of components that has shown good adjuvant activities. Among them, β-sitosterol is known to increase Th1-related cytokines, lymphocytes proliferation and greater NK cells activity [23].

The development of new adjuvants is, like vaccines developments, not an easy process [24]. In addition, difficulty in technical preparation, modest stability and elevated costs of production are other parameters to take in consideration when an adjuvant must be chosen.

Components extracted from natural product (e.g. flavonoids and vitamins) have immunomodulating and immunostimulating properties. These substances can then represent a class of new and potentially effective adjuvants even because of their low toxicity and easy development [25]. Due to their minimum side effects, their easy extraction, their low production costs and their proven immune stimulating activity, citrus-derived molecules, as flavonoids, are potential candidates to be administered as adjuvants in a vaccine formulation against Hpv.

Flavonoids have well-known antioxidant properties like inhibition of enzymes that promote reactive oxygen species (ROS) formation, scavenging of ROS and upregulation of antioxidant defences. They also provide anticancer and antiviral activity. Talking about Hpv, oxidative stress is a cofactor required for malignancy progression; cigarette smoke and chronic inflammation increase this condition and are associated with persistent infection. During cervical carcinogenesis, oxidative DNA damage is shown by progressive increase of 8-Oxo-2′-deoxyguanosine levels and proteins oxidation in keratynocytes [26]. A lot of antioxidants have been connected with Hpv and new therapies, based on their use, have been suggested as pre-treatment or support treatment in association with chemotherapy [27–29].

Sweet oranges and lemons are rich in flavonoids, mainly of Hesperidin. This flavonoid, thanks to its immunomodulatory activity, could potentially take part to vaccine formulation because of its promising role as an adjuvant. Belonging to the same class, Neohesperidin and Naringenin are also valid substances to take into consideration [30, 31].

With the goal to speed-up the identification of different candidate adjuvants, techniques based on In vitro and in vivo approaches have often been combined with specific in silico approaches [32–35]. This successful combination of interdisciplinary techniques represents, nowadays, one of the major advance in drug discovery [36, 37]. Additionally, each approach allows to study the biological phenomena of interest, both from a molecular, a cellular and a systemic point of view and to obtain multi-scale analysis.

Here we present an agent based model able to analyze the immune system response induced by adjuvants extracted from citrus, in the context of Hpv infection. The model simulates the biological scenario of all the entities populating the cervix and involved in the mechanism of defense against the virus. We simulated different vaccination protocols with different adjuvants to evaluate the artificially induced immune response and to predict the best combination in terms of adjuvant type, timing and dosage.

## Methods

### The starting point of the model: virtual screening approach

To narrow the identification of potential citrus-derived adjuvants candidates to be used in vaccine formulation against Hpv, we initially used virtual screening method starting from a set of molecules (identified through a deep primary literature exploration) present in the essential oil of orange peel. In detail, virtual screening methodology is based on computational techniques able to identify, using as a starting point a set of compounds, prospective ligands that could represent a specific biological target. There are two different approaches to make virtual screening: one technique is based the molecular features of the potential ligands that may act as activators or inhibitors. The other approach is based on structure-based virtual screening that can provide more reliable results as it analyzes each ligand affinity with its own biological target by means of a function that provides a score. However, it suffers from the high requested use of computational power.

Since the aim of this work is to identify activator of TLR4, the structure-based virtual screening of a library of compounds contained in the orange fruit extracts and plant flavonoids was used.

TLR4 plays a fundamental role in pathogen product recognition (such as LPS) and consequent activation of innate immunity. This specific family type receptor mediates the production of specific cytokines necessary for the development of effective immunity.

To this aim, we then downloaded three-dimensional structures from PubChem [38], followed by conformational analysis using the Boltzman Jump method implemented in AMMP software (http://nova.disfarm.unimi.it/cms/index.php?Software_projects:AMMP_VE) and improved by Mopac 2012 program (http://OpenMOPAC.net). Conformational search procedures investigate conformational space analyzing the torsion of the angles or relative displacements and orientations in molecular structures. Search procedures may be divided into two categories: systematic (deterministic) and stochastic (probabilistic) search procedures. As exhaustive systematic search of the entire conformational space is a very time consuming process, probabilistic Boltzmann Jump search can be used to reduce search time. In Boltzmann Jump, the torsion angles of a molecule are randomly altered within a specified angular window using Metropolis algorithm to explore conformational space for energy minima. The Metropolis–Hastings algorithm is a Markov chain Monte Carlo (MCMC) method for obtaining a sequence of random samples from a probability distribution for which direct sampling is difficult.

As we were interested in stimulating TLR4, the crystal configuration of the mouse TLR4/MD-2/LPS complex was downloaded from the Protein Data Bank (PDB ID 3VQ2) and enriched with hydrogens, fixing the atom charges using the Gasteiger – Marsili method [39] and the CHARMM 22 potentials for proteins [40], using the characteristics built-in in VEGA ZZ package [41]. The application of NAMD 2.9 [42] allowed to optimize the model in order to decrease the high-energy steric interactions. The final step consisted in the LPS removal from the complex to generate the pocket needed to recognize LPS-mimetics by virtual screening computations.

Taking into account the best virtual screening scores for candidate adjuvants, we chose the best two citrus-derived adjuvants that potentially could take part in vaccine formulation against Hpv: Neohesperidin and Naringenin. Table 1 shows all the virtual screening evaluated candidate adjuvants.

**Table 1:** Candidate adjuvants virtual screening marks

Adjuvants	Score
Neohesperidin	−95.09
Naringenin	−87.23
Ruthin	−86.16
Pectolinarigenin 7-glucoside	−83.25

### The agent-based Hpv model

To help in establishing a vaccine formulation against Hpv sustained infections that owns, at least, the same degree of efficacy of the Gardasil with alum derived adjuvants, we designed a NetLogo agent-based model to test and predict the induced immune response of citrus-derived adjuvants. [43].

The model uses a grid of 25 × 25 cells (namely patches) to simulate a small portion of cervix epithelium. We used a time-step of Δ(t) = 1 h. We take into account all the important entities and their properties (cells, molecules, cytokines and interactions) that are recognized as essential to the dynamics of HPV infection. IgG levels were used as biomarker to determine the efficacy of the adjuvants. The introduction of all agents inside the simulation space is done using stochastic pulse trains instead of Gaussian white noise. Pulse trains can be described as impulses, usually represented by non-sinusoidal waveforms similar to square waves. In stochastic pulse trains, the period that occurs between two consequent impulses is not fixed but stochastic. In our model the pulse duration is very small, as we can have, at most, no more than one impulse per time-step. Stochastic pulse trains are used in our model for introducing new agents since, as suggested by Wu and Zhu [44], the introduction of agents using stochastic impulses is advisable in order to gain more realistic and general understanding of the effect of environmental fluctuations, leading to extinction of the species.

Taking into account the dynamics of the infection, we included into the model the following entities:

- *Keratinocytes (Kcs)* denote the main target of Hpv infection.

Kcs have two variables: energy and life. Energy is used to determine a state of “compliance” of the cells towards the infection. In fact, even if the virus reaches the epithelium, not all the cells let the virus enter. When Kcs are created inside the simulation space, each of them takes a random energy value (within the range) and if this value is less than 80, the cell becomes susceptible to the virus. Energy level can be chosen in the range 0–100. Its default setting is 100. Kcs used to take 3 weeks to go from the basal layer to the upper layer in which they desquamate and die, so 21 days are set as lifespan of Kcs. Infected Kcs, if not recognized by the immune system effector cells, are subject to virus genome integration in the nucleus with subsequent possible triggering mechanisms that lead to cancer sprout.

Dendritic Cells (DCs): DC are used to represent APCs activity i.e., promote T cell response through the capture and the presentation of antigens. *This family of agents has* only *the* life parameter.

These kind of cells, also called Langherans Cells (LCs), express TLRs, stimulate CD8+ T cells with IL-15 and produce IL-1α, TGF-β, IL-10, IL-12, GM-CSF, IL-6 and IL-8. In addition, they have the specialized role to secrete type I IFN and inflammatory mediators.

Specific events, such as death and reproduction, govern the number of these entities over time. The procedure for these entities consists in simulating innate immunity by taking contact with Hpv: if one Hpv agent moves and stays in the same patch in which a DC is located at the same time-step, the DC is stimulated to produce a molecule of interferon. Additionally, when a DC interacts with Hpv, it modifies its state to “MHC II presenting”. DCs that change their state according to this described process, represent those cells that have endocytosed, digested within lysosomes, processed the virus and have loaded onto MHC class II molecules the resulting epitopes fragments. This complex migrates to the cell surface ready to mainly interact with immune cells, like T-helper cells. T-helper cells then help to trigger an appropriate immune response, like localized inflammation due to recruitment of phagocytes or antibody response by activation of B cells.

- *Natural killer cells (NK cells)*: this type of agents appear inside the model after a few time steps. This time lag indicates the time required to these cells to be recruited.

NK cells are stimulated by type 1-IFN and cytokines like IL-12 and IL-18. These cells are important components of the innate immune system, capable of killing infected cells by granule cytotoxicity that leads to the apoptosis of the target. Like DCs, they have only the life parameter.

NK cells move around the grid and their role is to catch and destroy infected Kcs. This happens when an infected Kcs stays in the radius of a NK that, recognizing its infection state, kills it. The radius can be managed in the interface. NK activity is regulated by another parameter, called “nk_downregulation”. This variable can be set to a specific value and acts as reference in a probabilistic evaluation of the activity. A random number is generated and, if it is less than the value of the variable, the NK kills the cell.

- *Interferon (IFN)*: this *type of agents* does not initially populate the world, but it sprouts only if one Hpv *agent* moves and stays in the same patch in which a DC *agent* is located.

The DC is stimulated to produce a molecule of interferon; these molecules are modelled because of their antiviral, antiproliferative and immunostimulatory properties. In this case, they provide an antiviral state that prevents cells to be infected or blocks intracellular viral mechanism that lead to precancerous formations. Being molecules, they do not have any procedure referred to reproduction and they live long as the lifespan set.

Their activity is regulated, like NK cells, by the parameter “ifn_downregulation”. This variable is set to a specific value and gives a reference for probabilistic evaluation of the activity. A random number is generated and, if it is lesser than the value of the variable, the IFN will bind to an infected Kcs in the same patch, letting it to return the health state.

- *Cytotoxic T cells (CTLs)*: these *agents* are not initially present into the simulation space. Their appearance in the model happens after a few ticks.

If exposed to infected cells, CTLs release the cytotoxins perforin, granzymes, and granulysins that lead to apoptosis of the target. A second way to induce apoptosis is via cell-surface interaction between the CTL and the infected cell. CTL expresses the surface protein Fas ligand, which can bind to Fas molecules expressed on the target cell.

Unlike others entities, each time a CTL kills an infected Kcs, it is stimulated to add another agent of its own type. They have only the life parameter. A variable called “ccl20” controls this mechanism in the same stochastic way described above for NK or IFN. The variable is called “ccl20” referring to the cytokine that has strongly chemotactic activity for lymphocytes.

- *B Cells (B): B lymphocytes are modelled because they are the effectors of the humoral immunity component of the adaptive immune system by secreting antibodies.*

B cells could be found both beneath the basement membrane, in the dermis, and over the epidermis, in the mucosal layer rich in innate and adaptive immune agents, so these cells are present in the grid at time zero.

Like for the previous agents, B cells have a lifespan, move, die and reproduce themselves as in the real biological scenario. Their activity is triggered by the presence, in their radius, of a MHC II presenting Dendritic cell, implying the interaction with T helper cells. The outcome of the B cell activation is the production of two types of B cells: “memory B cells” and “plasma B cells”. The former quadruples its lifespan and, if it meets a Hpv agent in the same patch, it produces Immunoglobulins G without requiring further activation. Plasma B cells keep instead the same lifespan of the progenitors and have the major function to produce immunoglobulins G.

- *IgG*: they are released by both memory and plasma B cells.

The model takes into account the number of IgG, as it is the main type of antibody found in blood and extracellular fluid. IgG protects from virus infection through several mechanisms like agglutination and opsonisation of the antigens, allowing their recognition by phagocytic immune cells, it activates the classical pathway of the complement system and it also plays an important role in antibody-dependent cell-mediated cytotoxicity (ADCC). All these events lead to extracellular neutralization of the virus, indeed they work until the pathogen has not entered the cells or when the Kcs, at the time of their maturation, desquamate and release new virions that can be caught. The presence of adjuvants influences the number of these entities.

- *Regulatory T cells (Tregs)*:

Tregs are a population of T cells that modulate the immune system response, maintain tolerance to self-antigens and prevent autoimmune diseases. Tregs have immunosuppressive properties and downregulate activation and proliferation of effector T cells. In our model, they are modeled because APC-mediated activation leads to production of IL-10 and TGF-β that downregulate CTLs. Their only parameter is “life”. Their role is to catch CTL agent and ask them to become inactivated, when they are in the radius. Every time a Treg inactivates a CTL, it is stimulated to add another agent of the same type.

- *Hpv*: This agent owns the “life” parameter only.

In natural infection, the virus requires about 4 h to enter the cell, so in the simulation, viruses move on the grid and, after four ticks, if they are in the same patch with a Kcs that has an energy that is less than 80 (that indicates low energy and “compliance”), they set them in an infected state.

If, after four time-steps, all the infection requirements are not fulfilled, the virus disappears and the Kcs remains healthy, suggesting an unsuccessful pathogenic attack.

**Fig. 1:**
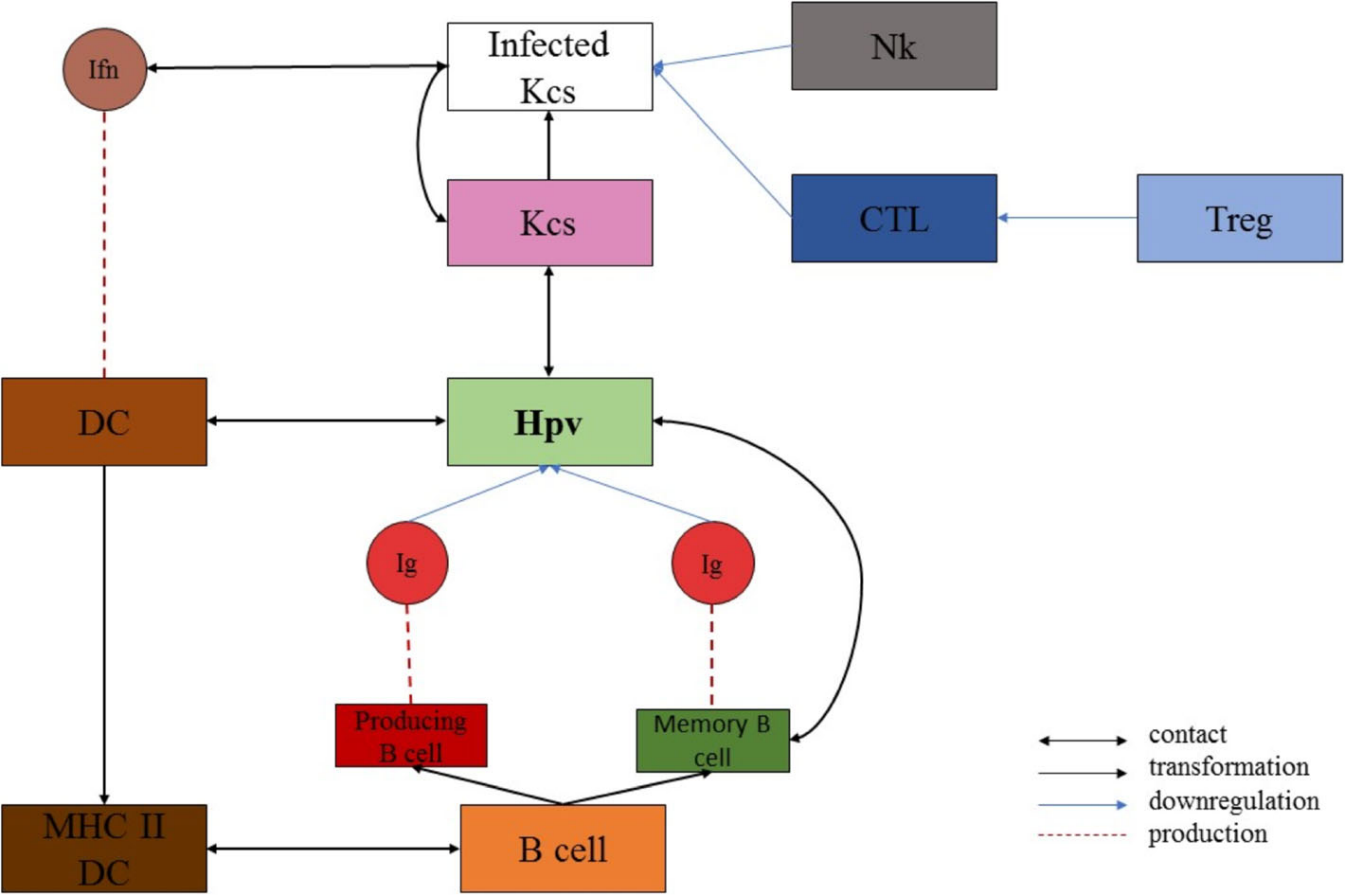
Graphical representation of the NetLogo model. The flow is the following: *i)* Hpv let Kcs change their color and state: from pink/healthy to white/infected; *ii)* DC processes Hpv antigen becoming MhcII-presenting DC and produces IFN; *iii)* IFN acts on infected Kcs bringing them back to healthy state; *iv)* MhcII-presenting Dc activates B cell; *v)* B cells become both memory B cells and plasma B cells; *vi)* Memory B cells are stimulated by Hpv to differentiate into IgG-producing plasma B cells; *vii)* Plasma B cells release IgG; *viii)* IgG catches and kills Hpv; *ix)* NK kills infected Kcs; *x)* CTL kills infected Kcs; *xi)* Treg downregulates CTLs

During the simulation time, the virus moves on the grid, it could be caught and killed by IgG, it could activate DCs and it could stimulate B cells to release IgG. Figure 1 depicts the modeled biological scenario.

Finally, we modeled the two candidate adjuvants with the best scores resulting from virtual screening analysis.

Neohespheridin and Naringenin were introduced in combination with viral antigen particles (“hpv” agents) to simulate a vaccine injection. Neohesperidin at concentration of 10 μg, Neohesperidin at concentration of 1 μg and Naringenin at concentration of 1 μg, are agents named, respectively, “adj1”, “adj2” and “adj3”.The integration of these entities allows to investigate the final levels of IgG production promoted by B cells.

Such agents are placed at random on the simulation space in combination with the viral antigens when a vaccine injection is done. As already stated, their role is to promote the activation of the immune response. To this end, if an adjuvant is in the same position with a plasma B cell, such cell will be further stimulated to produce antibodies with a given probability.

This interaction probability varies from an adjuvant to another, and has been set according to the virtual screening scores predicted by the virtual screening procedure (i.e., the better is the scoring, the higher is the probability of interaction).

**Fig. 2:**
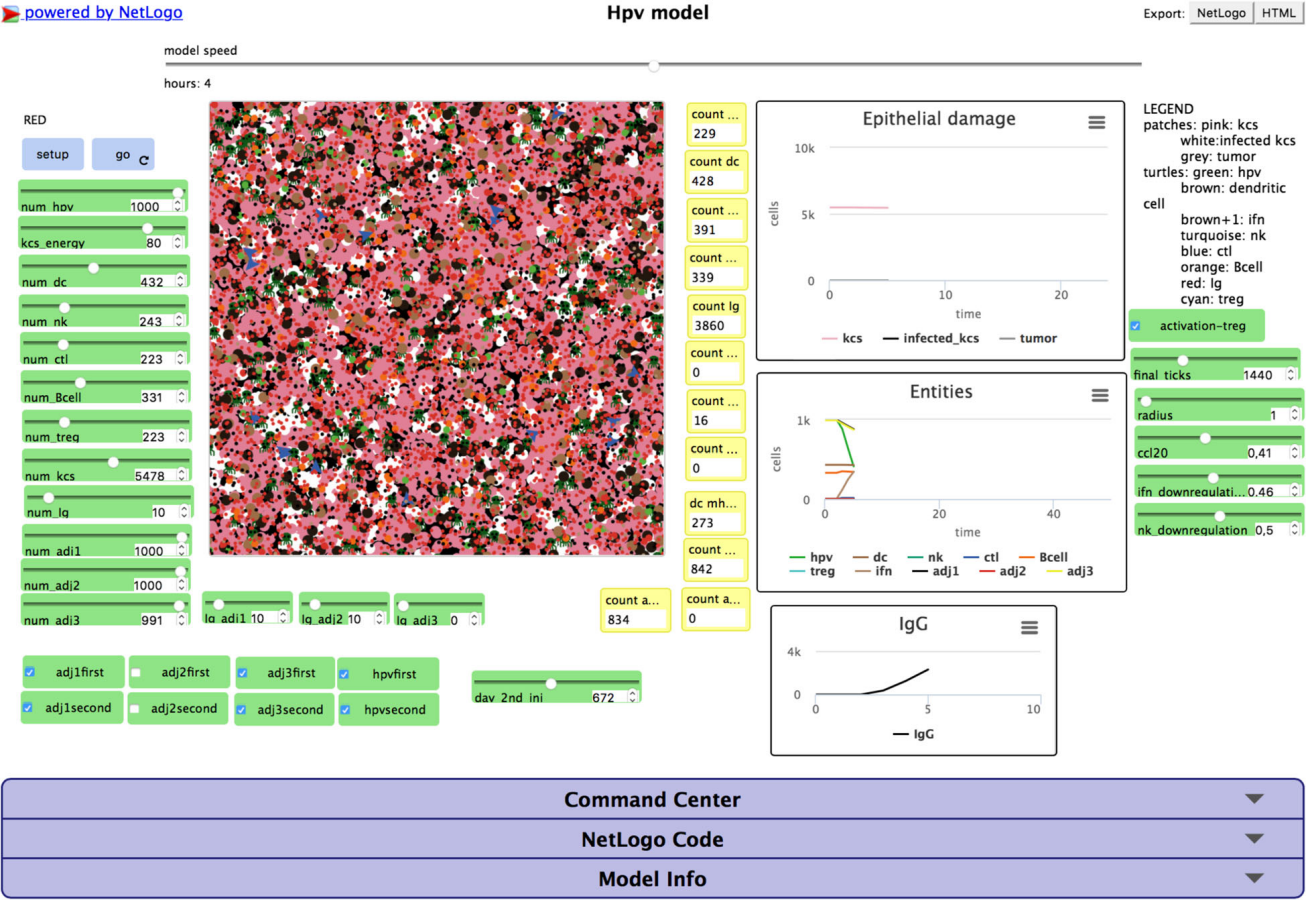
Screenshot of the web interface of the Hpv model. The HPV NetLogo web interface. The two cyan buttons “setup” and “go” allow to set-up and start the simulation. The green boxes contain the sliders and the check buttons that allow to modify the simulation parameters. The central box shows the simulation space with the involved entities. The yellow boxes allow to visualize the actual number of entities as the simulation advances. The three real-time graphs show the Epithelial damage, entities, and IgG levels. On the bottom, the three slidedown windows allow to interact and modify the model behavior, as command center window that allows to put real-time commands, or the NetLogo code window that allows to show, modify and recompile the code

A screenshot of the web interface of the model (available at http://www.francescopappalardo.net/Hpv-Adj-Model/) is presented in Fig. 2.

### Simulation settings

Each simulation represents a protocol of immunization that requires two vaccine administrations, the first always administered at day 0 and the second administered at day 14 or at day 21 or at day 28. We also evaluated IgG levels concentration in the presence of different adjuvants combinations.

Adjuvants activity was measured observing the total number of IgG produced and IgG concentration/time behavioral curves. Adjuvants that show high rates of IgG concentration and long-term protection guarantee an optimal program of immunization. The conducted experiments are summarized in Table 2. Simulation parameters are described in Table 3.

**Table 2:** Simulation experiments performed through NetLogo framework. We limited the in silico testing of the vaccination protocols only to them that are free form possible side-effects (as highlighted from preliminary safety in vivo testing) and that showed in silico the best humoral response evaluated by the IgG titers dynamics

Test	Adjuvants	Day of administration
a	adj1	0 and 14
b	adj1	0 and 21
c	adj1	0 and 28
d	adj2	0 and 14
e	adj2	0 and 21
f	adj2	0 and 28
g	adj3	0 and 14
h	adj3	0 and 21
i	adj3	0 and 28
l	adj1 + adj2	0 and 14
m	adj1 + adj2	0 and 21
n	adj1 + adj2	0 and 28
o	adj2 + adj3	0 and 14
p	adj2 + adj3	0 and 21
q	adj2 + adj3	0 and 28
r	adj1 + adj3	0 and 14
s	adj1 + adj3	0 and 21
t	adj1 + adj3	0 and 28

**Table 3:** Simulation parameters. Quantities are the ones that are initially proposed when the simulation framework is launched for the first time

Parameter	Cells/mm^3	Parameter	Molecules/mm^3	Parameter	Arbitrary Unit
num_hpv	1000	num_adj1	1000	kcs_energy	80
num_dc	432	num_adj2	989	final_ticks	1440
num_nk	243	num_adj3	991	radius	1
num_ctl	233	Ig_adj1	10	ccl20	0.41
num_Bcell	331	Ig_adj2	10	ifn_downregulation	0.46
num_treg	223	Ig_adj3	0	nk_donwregulation	0.60
num_kcs	5478	num_Ig	10	day_2nd_inj	672

### Hpv preparation and characterization

HPV16L1 gene codon optimized for yeast expression was cloned into pPICZa vector and expressed in Pichia Pastoris KM71 strain. Protein expression in selected clones was confirmed by western blotting with specific mouse monoclonal antibodies (Abcam) against HPV16. Master and working cell banks were prepared in yeast peptone dextrose media, whereas routine production batches were produced in chemically defined synthetic media by batch fermentation in 3 sub-stages. The cells were induced by methanol for protein expression. The batch was harvested, pelleted, lysed by a high-pressure homogenizer at 28 kpsi, and clarified. The clarified lysate was loaded onto cation exchange resin to purify the HPV16 L1 antigens and sterile-filtered in a 0.2 μm filter. Final concentrates were stored at 2–8 °C till use. Total protein content was assessed by BCA method and antigen purity, not less than 95%, was determined by SDS PAGE Coomassie stained. Protein identity was determined by Western Blot analysis. To assess HPV16L1, Virus Like particles purified protein were negatively stained with 1% uranyl acetate and examined under electron microscope.

### Mice and immunizations

Immunization experiments were performed in Balb/c female mice (Envigo). Mice were accommodated in suitable animal care facility and treated in accordance with EU guidelines. Balb/c mice were randomly distributed in groups (5 mice per group) and marked according to Table 4. All groups, except group CTRL that received 1 μg/dose of HPV16L1 at time 0 and 14, were immunized as shown in Table 4. Animals belonging to the groups A to D, received each dose of HPV16L1 formulated with Naringenin or Neohesperidin as adjuvant. For each tested adjuvant except for Naringenin, two different concentrations were tested at 1 or 10 μg/dose. Mice euthanized and blood collection were done at 35 days after the first immunization and sera samples were kept at −20 °C till use. Supervision and weight recording of the mice were done through the whole experiment.

**Table 4:** In vivo experiments summary. NH stands for Neohesperidin while NAR stands for Naringenin

Group	#of mice	Treatment (days of administration)
CTRL	5	HPV16L1 only (0 and 14)
A	5	HPV16L1 + NH 10 μg + NH 1 μg (0 and 14)
B	5	HPV16L1 + NH 10 μg + NH 1 μg (0 and 21)
C	5	HPV16L1 + NH 10 μg + NH 1 μg (0 and 28)
D	5	HPV16L1 + NH 10 μg + NAR 1 μg (0 and 28)

### ELISA setting

Ninety-six wells plates (Costar) were coated overnight at 4 °C with 100 μl per well of a 10 μg/ml solution of HPV 16 L1 in Na2CO3 0.05 M pH 9.6. Wells were then blocked with 200 μl per well of 10% dry milk in PBS solution for 1 h at 37 °C, followed by one wash with PBS. Plates were then incubated with serial dilutions of the mouse serum in PBS containing 0.05% Tween 20 and 3% dry milk for 1 h at 37 °C. After being washed three times with PBS containing 0.05% Tween® 20, plates were incubated with HRPconjugated goat anti-mouse IgG, IgG1 or IgG2a antibody (Sigma-Aldrich) for 1 h at 37 °C. After being washed three further times, 100 μl TMB-substrate (Termo Fisher) was added, and the plates were incubated in the dark at room temperature for 15 min. The reaction was stopped by addition of 100 μl 1 M H2SO4 and optical densities (OD) were read at 450 nm using a Victor V (Perkin Elmer).

## Results and discussion

From virtual screening platform we selected the best ranked scores for Neohesperidin and Naringenin and they are respectively −95.09 and −87.23 (the lower is the score, the better is the docking).

**Fig. 3:**
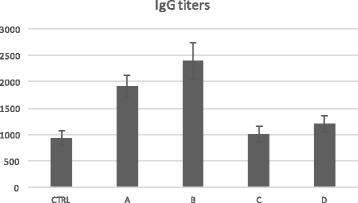
In silico results of the best vaccination protocols obtained by our computational model. Antibodies levels are expressed in arbitrary units while time is expressed in hours. Line A shows IgG levels detected after the administration of 10 μg of Neohesperidin at day 0, followed by a second injection of 1 μg of Neohesperidin at day 14, corresponding to the “t” in silico experiment of Table 1. Line B, IgG levels detected after the administration of 10 μg of Neohesperidin at day 0, followed by a second injection of 1 μg of Neohesperidin at day 21. This vaccination protocol corresponds to the “m” in silico experiment of Table 1. Line C depicts IgG levels recorded after the administration of 10 μg of Neohesperidin at day 0, followed by a second injection of 1 μg of Neohesperidin at day 28, corresponding to the “n” in silico experiment of Table 1. Finally, line D shows IgG titers recorded after the administration of 10 μg of Neohesperidin at day 0, followed by a second injection of 1 μg of Naringenin at day 28. This vaccination protocol corresponds to the “l” in silico experiment of Table 1. CTRL line corresponds to the control case i.e., HPV16L1 only, administered at time 0 and 14

According to the in silico simulation experiments, we selected the best four optimal vaccination protocols against Hpv infection. The best vaccination protocols are represented by “l”, “m”, “n” and “t” in silico experiments, as described in Table 1 and their relative antibodies levels are depicted in Fig. 3, respectively in line A, B, C and D. The first vaccination protocol (A) consists of the combination of “adj1” (Neohesperidin at the dosage of 10 μg) and “adj2” (Neohesperidin at the dosage of 1 μg), respectively administered at day 0 and day 14 (time step = 336). The mean value of IgG distribution obtained is 425,737. The second vaccination protocol (B) refers to the combination of adj1 and adj2 (Neohesperidin at the dosage of 1 μg and Neohesperidin at the dosage of 10 μg), respectively administered at day 0 and 21 (time step = 504). The mean value of IgG distribution obtained is 443,873. The third vaccination protocol (C) denotes the combination of adj1 and adj2, respectively administered at day 0 and 28 (time step = 672). The mean value of IgG distribution obtained for this vaccination protocol is 380,650. The last vaccination protocol shown in panel D consists of Neohesperidin at the dosage of 10 μg and Naringenin at the dosage of 1 μg, administered at day 0 and at day 28 (time step = 672). The mean value of IgG distribution is 399,723.

According to the in silico predictions, the administration protocols showed very different behaviors. While protocols A and B showed a very similar response in the number of IgG, with initial higher peaks, protocols C and D showed IgG levels that were, at least in the initial phase, somewhat similar to the control response. To gain a long-term protection it is mandatory to stimulate the immune system enough in order to entitle the production of memory B cells. Both higher peaks in the number of IgG and the total number of IgG in time may be considered as possible indicators of good and sufficient acquired immunity. To this end, we calculated the L2-norm (Euclidean norm) on the IgG numbers obtained with the different protocols. This norm computes the square root of the sum of the squares of IgG levels over time. Such an indicator may represent a good measure of the quality of a protocol because, by construction, it will both favor higher IgG peaks without forgetting the total quantity of IgG over time. In Table 5, we summarized the L2- norm values for all the protocols tested in silico.

**Table 5:** L2-norm values computed for each tested in silico protocol. The values were sorted from the biggest to the smallest. Groups l and m (respectively A and B) resulted the best ranked

Group	L2-norm values
l	22,688,214.95
m	21,602,770.65
t	17,159,145.29
n	16,701,542.87
s	16,444,521.25
o	16,437,544.43
r	15,988,501.39
p	14,524,402.5
a	13,966,799.75
c	13,492,741.89
e	12,881,435.2
d	12,646,115.38
b	12,619,069.36
g	12,481,577.26
q	11,745,594.69
f	10,452,779.34
h	10,261,408.85
i	9,067,796.302

**Fig. 4:**
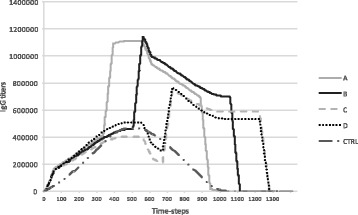
In vivo results. Balb/c mice subdivided in five groups of five individuals were used. The control group (CTRL, the first) received two administration of HPV16L1 at time 0 and 14; The second group (A) is cured with the Neohesperidin at dosage of 10 μg, followed by Neo-hesperidin at dosage of 1 μg, administered, respectively at day 0 and 14. The third group (B) received Neohesperidin, administered at a dosage of 10 μg followed by Neo-hesperidin at dosage of 1 μg, inoculated, respectively at day 0 and 21. The forth group (C) get Neohesperidin at dosage of 10 μg followed by Neo-hesperidin at dosage of 1 μg, administered, respectively at day 0 and 28. The last group (D) is cured with the Neohesperidin at dosage of 10 μg followed by Naringenin at dosage of 1 μg, administered, respectively at day 0 and 28. The total duration of the experiments was 35 days. Subsequent in vivo experiments validated the predictions made by the in silico simulation framework

The L2-norm was higher for protocols l and m (A and B respectively in Table 3B), thus suggesting that these candidate protocols may be the best ones for acquiring immunity. As one can appreciate looking at Fig. 4, in vivo experiments confirm the in silico predictions as protocols A and B entitled best IgG titers. The L2-norm seems to be a good method to evaluate the protection conferred by vaccination protocols both in silico [45] and in vivo.

## Conclusions

Novel vaccines that are almost based on subunit antigens are often characterized by a inadequate immunogenicity when administered alone. Therefore, the discovery of new adjuvants that can overcome this limited immunogenicity are urgently desirable. Unfortunately, nowadays there are only few licensed adjuvants approved for human use, that are almost based on Aluminium mineral salts. These adjuvants, that possess a good safety and efficacy, however, do not guarantee a good degree of immune response when used in combination with small peptides. Adjuvants extracted form natural products offer a remarkable immune system stimulation with reduced side effects.

A good number of approaches based on both in silico and in vivo techniques are present in the biotechnology market. They provide a way to envisage possible adjuvants candidates without, however, offer a methodology to analyze and quantify the immune system dynamics as a whole.

In this paper, we developed a model that combines the results coming from a virtual screening approach, used to preselect promising citrus derived compounds, with an agent based model that reproduces HPV induced disease and relevant involved immune system entities. This “multi-scale” approach was able to predict the dynamics of the immune response induced by several vaccination formulations against the HPV16 virus. Finally, in vivo testing was in a good agreement with the predicted results.
